# Optimization Design and Simulation of a Multi-Source Energy Harvester Based on Solar and Radioisotope Energy Sources

**DOI:** 10.3390/mi7120228

**Published:** 2016-12-14

**Authors:** Hao Li, Gaofei Zhang, Zheng You

**Affiliations:** Collaborative Innovation Center for Micro/Nano Fabrication, Device and System, State Key Laboratory of Precision Measurement Technology and Instruments, Department of Precision Instruments, Tsinghua University, Beijing 100084, China; hao-li12@mais.tsinghua.edu.cn (L.H.); yz-dpi@mail.tsinghua.edu.cn (Y.Z.)

**Keywords:** multi-source, energy harvester, solar, radioisotope battery

## Abstract

A novel multi-source energy harvester based on solar and radioisotope energy sources is designed and simulated in this work. We established the calculation formulas for the short-circuit current and open-circuit voltage, and then studied and analyzed the optimization thickness of the semiconductor, doping concentration, and junction depth with simulation of the transport process of β particles in a semiconductor material using the Monte Carlo simulation program MCNP (version 5, Radiation Safety Information Computational Center, Oak Ridge, TN, USA). In order to improve the efficiency of converting solar light energy into electric power, we adopted PC1D (version 5.9, University of New South Wales, Sydney, Australia) to optimize the parameters, and selected the best parameters for converting both the radioisotope energy and solar energy into electricity. The results concluded that the best parameters for the multi-source energy harvester are as follows: Na is 1 × 10^19^ cm^−3^, Nd is 3.8 × 10^16^ cm^−3^, a PN junction depth of 0.5 μm (using the ^147^Pm radioisotope source), and so on. Under these parameters, the proposed harvester can achieve a conversion efficiency of 5.05% for the ^147^Pm radioisotope source (with the activity of 9.25 × 10^8^ Bq) and 20.8% for solar light radiation (AM1.5). Such a design and parameters are valuable for some unique micro-power fields, such as applications in space, isolated terrestrial applications, and smart dust in battlefields.

## 1. Introduction

With the rapid development of the micro-electro mechanical system (MEMS), the micro-power has been recognized as the key technology of MEMS. Among the various micro-powers being investigated, the betavoltaic radioisotope battery is a new type of power source which is advantageous in terms of a long working life, high reliability, flexibility in rugged environments, and being maintenance-free [1]. Researchers have succeeded in developing betavoltaic batteries with different kinds of semiconductor substrates (e.g., Si, GaN, SiC) and different kinds of radioisotope sources (e.g., ^63^Ni, ^3^H, ^147^Pm) over the past decade [2,3,4,5]. However, the output power and conversion efficiency of the betavoltaic battery are still rather low, which restricts its application. The Amit Lal Research Group of Cornell University developed ^63^Ni-Si batteries with a 0.32 nW output power and 0.3% conversion efficiency [6]. The Blanchard Research Group at the University of Wisconsin developed an inverted triangular straight-slot ^63^Ni-Si battery with a 0.6% conversion efficiency [7]. The Hang Guo Research Group of the Pen-Tung Sah Lab of Xiamen University developed ^147^Pm-GaN batteries with a 44 nW output power and increased the conversion efficiency to 0.767% [8]. Bower et al. designed the ^63^Ni-GaN Schottky battery which can achieve an energy conversion efficiency of 2.25% [9]. Compared with the betavoltaic battery, solar cells can provide more power. Deng et al. researched a passivated emitter and rear cell (PERC) solar cell which can provide a 20.8 mW/cm^2^ power density with a multicrystalline silicon substrate [10]; Masuko et al. achieved a 25.6 mW/cm^2^ power density with a rear junction crystalline Si solar cell [11]. Kayes et al. designed a single-junction GaAs solar cell with a power density as high as 27.6 mW/cm^2^ [12]. Although the power density of the solar cell is rather large, it completely relies on solar radiation. When the ambient environment is not illuminated by the sun, it can generate no electric power at all [13]. This defect becomes serious when the solar cell is applied in some unique micro-power fields, such as smart dust in battlefields. Smart dust refers to a cubic-millimeter-scale sensing and communication platform that can monitor environmental conditions in both military and commercial applications [14,15]. Researchers at the University of Michigan developed a sophisticated 1 mm three-scale smart dust in 2012 [16,17]. Each layer measures less than 2.21 mm × 1.1 mm, and the height of the entire system is 0.4 mm, resulting in a 1.0 mm^3^ system. The power consumption ranges from 11 nW in sleep mode up to ~40 μW in active mode. A 0.54 mm^2^ solar cell is fabricated and a 0.6 μAh thin-film Li battery is included in the system. When the ambient environment is illuminated by the sun, the solar cell can power the system and charge the Li battery with 40 nW power. However, when the light disappears, the thin-film Li battery can only support the system in active mode for 8 min and in sleep mode for up to 2.3 days. When the power is completely consumed, the smart dust is disconnected. In consideration of the incubation period that the smart dust needs in battlefields, a long life and stable energy source are required to make sure the device can be awakened at any moment. Multi-source energy harvesters based on solar and radioisotope energy sources are the best choice for this problem. In order to solve the problem of the absence of a light source for solar cells and increase the output power of betavoltaic batteries, we developed a novel multi-source energy harvester. The proposed harvester can convert solar energy and radioisotope energy into electrical power with a single chip.

In this research, based on the basic principle of the betavoltaic effect and the theory of semiconductor physics, we established the calculation formulas for the short-circuit current and open-circuit voltage, and analyzed the optimization thickness of the semiconductor, the doping concentration, and the junction depth using the Monte Carlo simulation program MCNP (version 5, Radiation Safety Information Computational Center, Oak Ridge, TN, USA). In order to improve the conversion efficiency of the solar cell unit, we adopted PC1D (version 5.9, University of New South Wales, Sydney, Australia) to optimize the parameters, and the results concluded that the best parameters for the multi-source energy harvester are as follows: Na = 1 × 10^19^ cm^−3^, Nd = 3.8 × 10^16^ cm^−3^, a PN junction depth of 0.5 μm, and so on. Under these parameters, when the rear of harvester (1 cm^2^) is irradiated with the ^147^Pm radioisotope source (with the activity of 9.25 × 108 Bq), the short-circuit current, open-circuit voltage, output power, and conversion efficiency are 1.51 μA, 384 mV, 0.46 μW, and 5.05%, respectively. On the other hand, when the front of the harvester is illuminated by the sun (with an air-mass of AM1.5), the output power can increase up to 20.8 mW. Such power is sufficient for many low-power applications, such as smart dust and wireless sensor nodes.

## 2. Structure and Models

### 2.1. Similarities and Differences between Solar Cells and Betavoltaic Batteries

Several similarities exist in solar cells and betavoltaic batteries, which make it possible to develop a multi-source energy harvester based on solar and radioisotope energy sources. Firstly, the betavoltaic effect is similar to the photovoltaic effect as electron-hole pairs (EHPs) are produced when the PN junction semiconductor material is struck by photons or β particles. With the help of a built-in electric field, electrons move to the N region, while holes move to the P region, and then are collected by the electrode and flow out to the outside circuit. Secondly, the semiconductor materials to fabricate betavoltaic batteries and solar cells are nearly the same, and they include Si, GaN, GaAs, etc.; therefore, the betavoltaic battery and solar cell can be deployed on a mutual substrate. Thirdly, the fabrication progresses of the betavoltaic battery and solar cell are similar. The key step of the fabrication processes for both of them is the doping of the PN junction, which can be realized by oxidation diffusion and ion implantation. The key difference between them is the electron generation mechanisms. In a betavoltaic battery, a single particle from the radioisotope generates a large number of electron-hole pairs, and the spatial distribution of these electrons is different compared those from a solar spectrum. The penetration depth of the incident radiation, rather than the absorption depth, determines the distance over which the generated electrons need to be collected. In addition, another difference between the betavoltaic battery and solar cell is that the solar cell should take into account optical considerations (i.e., low reflection, etc.).

### 2.2. Structure and Principle of the Multi-Source Energy Harvester

**Figure 1:**
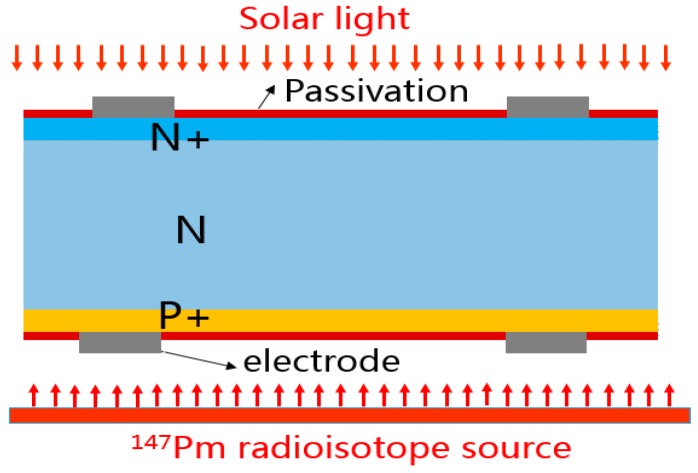
Schematic diagram of multi-source harvester.

As shown in Figure 1, the proposed multi-source energy harvester is designed with a bifacial N + NP + Si structure. The front of the harvester is illuminated by the sun, and makes the harvester convert solar light energy into electricity with a photovoltaic effect. The rear of the harvester is irradiated with the ^147^Pm radioisotope source. The emission of energetic β particles from the radioisotope irradiates the semiconductor PN junction, thus resulting in the generation of electron-hole pairs (EHPs) through ionization and excitation. The EHPs were generated within the depletion region, and swept across the PN junction by the depletion electric field into the external load to produce electrical power.

### 2.3. Equivalent Circuit Model and Formulas

**Figure 2:**
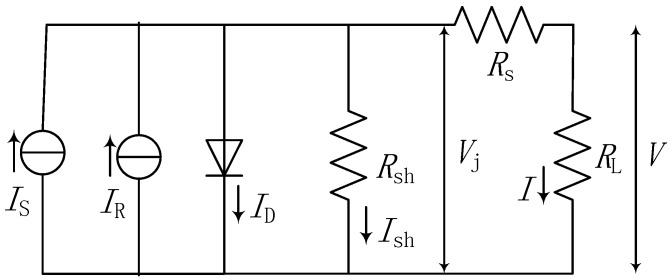
Equivalent circuit model of the multi-source harvester.

As the electrons induced both by photons and β particles move to the N+ region and the holes move to the P+ region, so the coexisting effects of the solar light energy source and the radioisotope energy source are equivalent for enhancing the ability of photons to produce EHPs or enhancing the ability of β particles to produce EHPs. When the solar light and radioisotope coexist, the current density is equivalent to the sum of the photon-induced current density and β particle–induced current density. Thus, the circuit model of the proposed multi-source energy harvester can be equivalent to that shown in Figure 2.

From its model, the short-circuit current, the open-circuit voltage, and the maximum output power can be derived as

(1) $$I_{\text{SC}}=(I_{R}+I_{S}-I_{0}(e^{\frac{qV_{j}}{kT}}-1))(\frac{R_{\text{sh}}}{R_{\text{sh}}+R_{S}})$$

(2) $$V_{\text{OC}}=\frac{KT}{q}\text{ln}(\frac{I_{\text{SC}}}{I_{0}}+1)$$

(3) $$P_{m}=FF\times V_{\text{OC}}\times I_{\text{SC}}$$

(4) $$FF=\frac{v_{\mathrm{oc}}-\text{ln}(v_{\mathrm{oc}}+0.72)}{v_{\mathrm{oc}}+1}$$

(5) $$\eta =\frac{P_{m}}{AqE_{\text{av}}}$$

where *I*_SC_ is the short-circuit current. *I*_R_ is the current generated by the radioisotope, *I*_S_ is the current generated by the solar light, *I*_0_ is the leakage current of the PN junction device. *R*_sh_ and *R*_S_ are the equivalent parallel resistance and series resistance of the harvester, respectively. *K* is Boltzmann’s constant, *T* is the absolute temperature, *v*_oc_ is the normalized open-circuit voltage which equals to *V*_OC_/(*nkT/q*). *P_m_* is the maximum output power, *FF* is the filling factor, *A* is the activity of the radioisotope, *E*_av_ is the average energy of the incident β particles, and *q* refers to the electron charge.

In this model, the solar light–generated current (*I*_S_) can be simulated by PC1D, but there is no special simulation software to calculate *I*_R_ directly. In previous studies, the calculation of *I*_R_ was primarily based on the theoretical model of Shockley on the semiconductor PN junction. This kind of analytical calculation process is too complex. In this research, we adopted MCNP to simulate the transport processes of β particles in semiconductor materials and to calculate the nuclear radiation–generated current *I*_R_. Theoretically, *I*_R_ can be derived as [18,19,20]:

(6) $$I_{R}=\int_{0}^{H}CE(x)\cdot q\cdot G(x)dx=\frac{qA}{\varepsilon }\int_{0}^{H}CE(x)\cdot E(x)dx=\frac{qA}{\varepsilon }\sum_{n=1}^{k}CE(n)\cdot E(n)$$

(7) $$CE(x)=1-\text{tan}\;h(x_{n}/L)$$

where *H* is the thickness of the semiconductor, *CE*(*x*) is the collection probability of electron-hole pairs, *q* is the electron charge, *E*(*x*) is the energy deposition, *E*(*n*) is the energy deposition of the Nth layer semiconductor calculated by MCNP, and *k* is the number of total layers in the semiconductor during MCNP calculation; *x_n_* is the distance of the *N*th layer from the depletion region, *L* is the minority carrier diffusion length, and tan*h* is the hyperbolic tangent function.

## 3. Simulation and Optimization Design

### 3.1. Energy Deposition in the Si Semiconductor

As shown in Equation (6), in order to calculate the nuclear radiation–generated current, we should obtain the energy deposition in the Si semiconductor first. The β particles emitted from the decay of the ^147^Pm radioisotope will lose energy during transportation. The energy deposition distribution and accumulated energy deposition of β particles can be simulated by MCNP.

MCNP is a general-purpose Monte Carlo N-Particle code that can be used for neutron, photon, electron, or coupled neutron/photon/electron transport. In this research, we first built up the geometric model of the multi-source energy harvester. In order to ensure the accuracy of the calculation, we divide the Si semiconductor substrate into several pieces, and then calculated the energy spectrum of the β particle energy emitted by ^147^Pm. With these data, we write up the S card, M card, and choose the simulation model to program the code.

**Figure 3:**
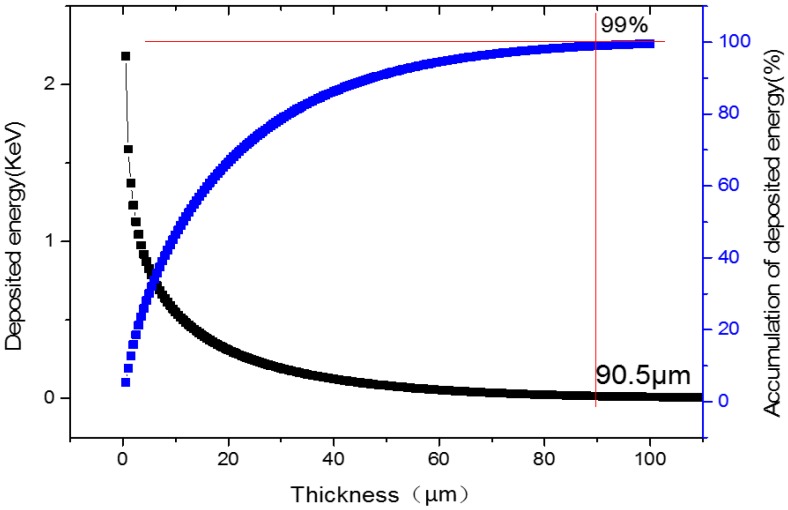
Energy depositions in Si for the ^147^Pm radioisotope.

Figure 3 shows the simulated results. From that figure, we can conclude that about 99% of the β particle energy emitted by ^147^Pm is deposited in a thickness of no more than 90.5 μm in the Si semiconductor. In addition, when the thickness of the Si semiconductor is decreased to 80 μm, the available power of the deposited energy will only be decreased by less than 1%. Based on the above analysis, a thickness of 80 μm to 100 μm is enough for depositing the β particle energy in the Si-^147^Pm radioisotope battery. Furthermore, in order to satisfy the request of converting the solar energy and radioisotope energy simultaneously, in this research, we increased the thickness of the Si semiconductor to 150 μm.

### 3.2. Doping Concentration Optimization

The doping concentration of the P-type and N-type regions decides the depletion region width, the intensity of the built-in field, the collection probability of EHPs, and the leakage current of the multi-source energy harvester. A low doping concentration will result in a longer minority carrier diffusion length and a wider depletion region width, and a higher collection probability of EHPs. However, a low doping concentration will result in an increase in the leakage current and a reduction in the open-circuit voltage as well. As the doping concentration affects many other parameters in this multi-source energy harvester, we make a numerical analysis and simulation of the efficiency for converting the radioisotope energy into electrical power using the mathematical analysis software MATLAB (version 7.13, Math Works, Natick, MA, USA) with Equations (1)–(7).

**Figure 4:**
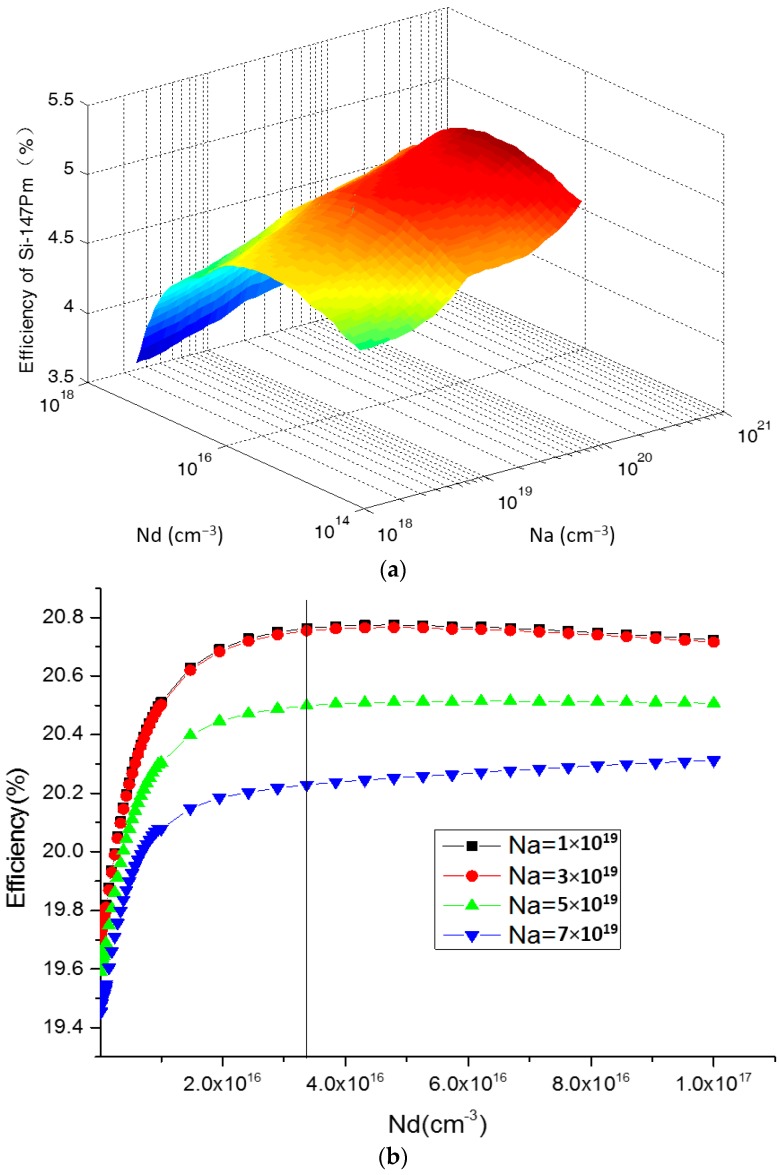
Relationship between the maximum conversion efficiency and doping concentration for the multi-source energy harvester. (**a**) Doping concentration parameters optimization simulated by MCNP (version 5, Radiation Safety Information Computational Center, Oak Ridge, TN, USA) for radioisotope energy harvesting; (**b**) Doping concentration parameters optimization simulated by PC1D (version 5.9, University of New South Wales, Sydney, Australia) for solar energy harvesting.

Figure 4 shows the relationship between the maximum conversion efficiency and doping concentration under the condition of 300 K, an activity of 9.25 × 108 Bq ^147^Pm radioisotope, and a 0.5 μm junction depth. From Figure 4a, we can conclude that heavy doping in the P+ region (Na about 10^19^ cm^−3^ to 10^20^ cm^−3^) and light doping in the N-type region (Nd about 10^16^ cm^−3^ to 10^17^ cm^−3^) can yield the highest efficiency for converting radioisotope energy into electrical power. To make further estimations of the performance for the multi-source energy harvester when it is under solar light illumination, we optimized Na and Nd by PC1D. In this simulation, the optimized parameters for radioisotope energy harvesting were used in PC1D, and set Na as 1 × 10^19^ cm^−3^, 3 × 10^19^ cm^−3^, 5 × 10^19^ cm^−3^, 7 × 10^19^ cm^−3^ separately, with Nd varying from 10^16^ cm^−3^ to 10^17^ cm^−3^. The simulated results are summarized in Figure 4b. From this figure we can conclude that the maximum efficiency for converting solar light energy into electric power is obtained when Na is about 1 × 10^19^ cm^−3^, and Nd is about 3.8 × 10^16^ cm^−3^.

### 3.3. Junction Depth Optimization

**Figure 5:**
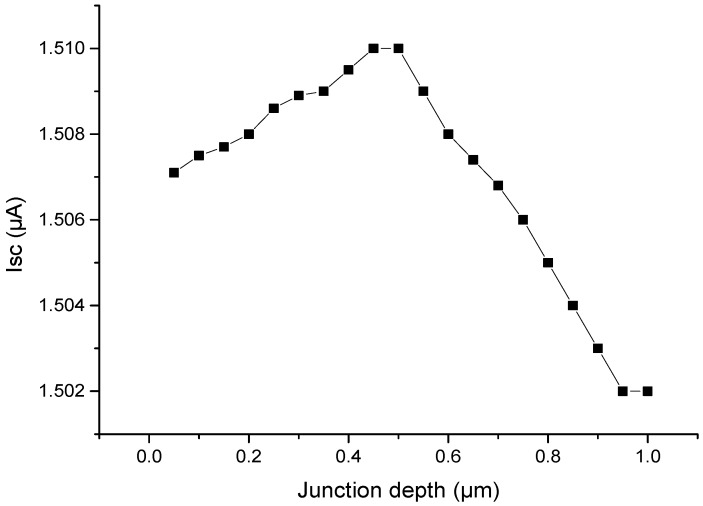
Relationship between *I*_SC_ and junction depth in Si–^147^Pm.

**Figure 6:**
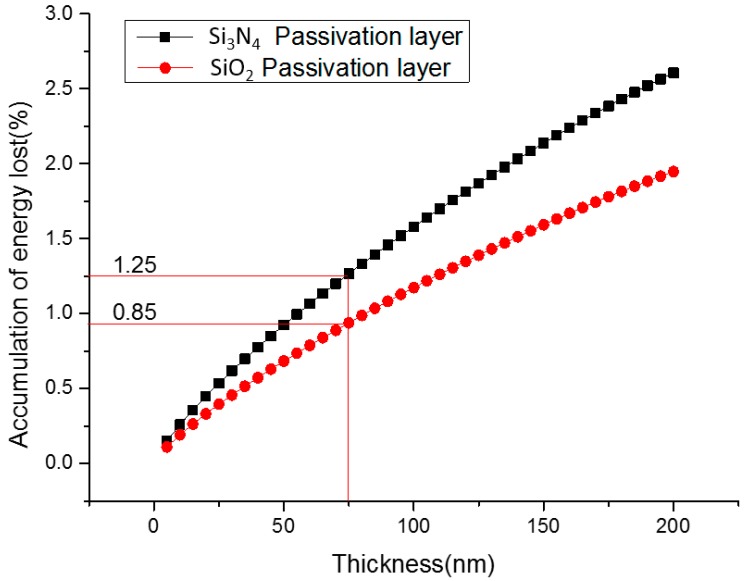
Radioisotope energy lost difference in the passivation layer with SiO_2_ and Si_3_N_4_ materials.

The junction depth affects the distance of the calculation layer from the depletion region, as shown in Equation (7). Therefore, it decides the collection probability of EHPs, and ultimately affects the short-circuit current. Figure 5 shows the relationship between the maximum short-circuit current and junction depth under the conditions of 300 K, activity of 9.25 × 10^8^ Bq ^147^Pm radioisotope, 1 cm^2^ junction area, Na of 1 × 10^19^ cm^−3^, and Nd of 3.8 × 10^16^ cm^−3^. From Figure 6, we can find that when the junction depth is about 0.5 μm, the harvester can obtain the maximum short-circuit current.

### 3.4. Passivation Layer Optimization

The surface of the Si semiconductor represents the largest possible disturbance of the symmetry of the crystal lattice and, hence, due to non-saturated bonds, a large density of defects within the bandgap exists at the surface of the crystal [21]. In order to avoid an unacceptably large efficiency loss caused by surface recombination, the passivation of the front and rear surfaces is needed for this harvester. Traditionally, the thermal growth of SiO_2_ is the most effective Si surface passivation technique for solar cells, and a PECVD layer of SiN*x* can also provide a nearly perfect passivation as well [22]. In this research, we analyzed the energy loss when β particles penetrate into the passivation layers (SiO_2_ and Si_3_N_4_) with the Monte Carlo simulation program MCNP. Figure 6 analyzes the relationship between the thickness of the passivation layer and the accumulated energy loss proportion in SiO_2_ and Si_3_N_4_ materials. From this figure, we can conclude that when the thickness of the passivation layer is constant, the energy loss in the SiO_2_ layer is lower than the Si_3_N_4_ layer. A 75 nm thickness of the SiO_2_ passivation layer will bring out an accumulated energy loss proportion of 0.85%, which is 0.4% proportion lower than that of the Si_3_N_4_ passivation layer with an equal thickness.

### 3.5. Simulation on Output Results

Based on the above analysis, we make a simulation of the output parameters of the proposed multi-source energy harvester for converting radioisotope energy and solar light energy into electricity with MATLAB and PC1D software. The conditions are listed as follows: temperature of 300 K, activity of 9.25 × 10^8^ Bq ^147^Pm radioisotope, junction depth of 0.5 μm, Na of 1 × 10^19^ cm^−3^, Nd of 3.8 × 10^16^ cm^−3^, 1 cm^2^ junction area, air mass of AM1.5. Table 1 shows the simulation results.

## 4. Conclusions

In summary, a novel multi-source energy harvester is designed and simulated in this research to increase the output power of betavoltaic batteries and to solve the problem of the absence of a light source for solar cells. We studied and analyzed the optimization thickness of the semiconductor, the doping concentration, the junction depth, and the material for the passivation layer. With these parameters, the proposed harvester can achieve a conversion efficiency of 5.05% for a ^147^Pm radioisotope source (with an activity of 9.25 × 10^8^ Bq) and 20.8% for solar radiation (AM1.5). Such a design and parameters are valuable for micro-power applications in space, isolated terrestrial applications, and smart dust in battlefields.

## Figures and Tables

**Table 1:** Simulated results with optimized parameters.

Energy Source	*V*_oc_	*I*_sc_	η	*P_m_*
Solar light (Front)	697 mV	37.7 mA	20.8%	20.8 mW
^147^Pm source(Rear)	384 mV	1.51 μA	5.05%	0.46 μW
